# Epidemiological characteristics an outbreak of ST11 multidrug-resistant and hypervirulent *Klebsiella pneumoniae* in Anhui, China

**DOI:** 10.3389/fmicb.2022.996753

**Published:** 2022-09-23

**Authors:** Zhien He, Weifeng Xu, Hang Zhao, Wei Li, Yuanyuan Dai, Huaiwei Lu, Liping Zhao, Changfeng Zhang, Yujie Li, Baolin Sun

**Affiliations:** ^1^Department of Oncology, The First Affiliated Hospital of USTC, Division of Life Sciences and Medicine, University of Science and Technology of China, Hefei, Anhui, China; ^2^College of Life Science and Technology, Mudanjiang Normal University, Mudanjiang, China; ^3^Department of Clinical Laboratory, Anhui Provincial Hospital of Anhui Medical University of China, Hefei, China; ^4^Clinical Laboratory Center, First Affiliated Hospital, Anhui University of Traditional Chinese Medicine, Hefei, China

**Keywords:** extended-spectrum β-lactamase, *Klebsiella pneumonia*, multidrug-resistant and hypervirulent *Klebsiella pneumoniae* (MDR-hvKp), virulence plasmid, whole genome sequencing

## Abstract

*Klebsiella pneumoniae* has become a primary threat to global health because of its virulence and resistance. In 2015, China reported multidrug-resistant (MDR) and hypervirulent *K. pneumoniae* (hvKp) isolates. The emergence of MDR-hvKp poses a significant threat to public health. We collected 76 MDR *K. pneumoniae* isolates from the same hospital, of which there were a total of six MDR-hvKp isolates. We performed multilocus sequence typing (MLST) and capsular typing, whole genome sequencing, comparative genome analysis, and phylogenetic analysis as well as phenotypic experiments, including growth curves, mucoviscosity assay, *Galleria mellonella* infection model, human whole blood survival, and human neutrophil bactericidal assay to further characterize the samples. We identified six large plasmids carrying extended spectrum β-lactamase (ESBL) genes or carbapenemase genes (*bla*_CTX–M–65_, *bla*_KPC–2_, *bla*_SHV–12_, *bla*_SHV–158_), 9 plasmids containing other drug resistance genes, and 7 hypervirulence plasmids carrying *rmpA* and *rmpA2* in ST11 MDR-hvKp isolates. Some of these plasmids were identical, whereas others differed only by insertion elements. In addition, we identified a plasmid, p21080534_1, that carries hypervirulence genes (*iucABCD*, *iutA*, *rmpA2*), a carbapenemase gene (*bla*_KPC–2_), and an ESBL gene (*bla*_SHV–12_), as well as MDR-hvKp 21072329, which did not carry *rmpA* or *rmpA2*, but exhibited hypervirulence and hypermucoviscosity. ST11 MDR-hvKp derived from hypervirulence and multidrug resistance plasmids not only causes significant treatment difficulties, but also represents an unprecedented challenge to public health. Therefore, urgent measures are needed to limit further spread.

## Introduction

*Klebsiella pneumoniae* is an opportunistic Gram-negative pathogen that causes various diseases including pneumonias, sepsis, and urinary tract infections (Podschun and Ullmann, 1998; Paczosa and Mecsas, 2016). Generally, *K. pneumoniae* colonizes the mucosal surface of the host without symptoms and primarily spreads through contact (Jarvis et al., 1985). For hosts with weakened immunity, such as the elderly, children, and critically ill patients, *K. pneumoniae* may result in serious hospital-acquired infections and community-acquired infections (CAI) (Paczosa and Mecsas, 2016). In recent years, infections caused by *K. pneumoniae* have steadily increased, particularly from multi-drug-resistant (MDR) strains, such as carbapenem-resistant *K. pneumoniae* (CRKP), extended spectrum β-lactamase (ESBL)-producing *K. pneumoniae*, and other MDR strains (Paterson et al., 2004; Munoz-Price et al., 2013). The US Centers for Disease Control and Prevention (CDC)’s 2019 report on antimicrobial resistance (AMR) listed Carbapenem-resistant Enterobacterales as a major threat (CDC, 2019); thus, the emergence of MDR *K. pneumoniae* has become a burden to the medical community.

Currently, the most widespread multilocus sequence types (MLST) of MDR *K. pneumoniae* include ST258, ST512, and ST11 (Ocampo et al., 2016; Moradigaravand et al., 2017; Wyres et al., 2020a). Although outbreaks caused by other types have been reported (Gorrie et al., 2018; Lowe et al., 2019), they are not as widespread. ST258 occurs predominantly in America and Australia (Kitchel et al., 2009; Kwong et al., 2018), ST512 is primarily located in Latin America (Mojica et al., 2012; Wyres et al., 2020a), and ST11 is most prevalent in Asia (Qi et al., 2011; Wyres et al., 2020b). In addition, ST258, ST512, and ST11 are distributed in various regions of Europe (Wyres et al., 2015; David et al., 2019).

In addition to its multidrug-resistant phenotype, *K. pneumoniae* is notorious for its virulence. In 1986, a case of purulent liver abscess brought hypervirulent *K. pneumoniae* (hvKp) into the public eye (Liu et al., 1986). Unlike classical *K. pneumoniae* (cKp, or non-hypervirulent strains), hvKp typically causes infection in healthy individuals, exhibits hypermucoviscous features, and produces higher capsule levels (Choby et al., 2020). The hypermucoviscous phenotype of hvKp usually results from *rmpA* and *rmpA2*, which are located on the hypervirulence plasmid (e.g., pK2044 and pLVPK) (Russo and Marr, 2019). In addition to *rmpA* and *rmpA2*, the metabolite transporter-encoding gene, *peg-344*, siderophore-encoding genes, *iroB* and *iucA*, are biomarkers of hvKp (Russo et al., 2018). The K-types of most hvKps are K1, K5, K20, K54, and K57, whereas the MLST are ST23, ST25, ST65, ST86, and ST375.

With the continuous spread of hypervirulence and MDR plasmids worldwide, some cKps acquired hypervirulence plasmids to form hypervirulent strains, whereas hvKps acquired MDR plasmids to form MDR hypervirulence strains. The K-types and MLST of hvKps are not limited to just a few types. In 2015, five cases of ST23-KL1 and ST1797-KL1 MDR-hvKps carrying *Klebsiella pneumoniae* carbapenemase (KPC)-2 occurred in the ICU of two hospitals in Zhejiang Province, China, which resulted in fatal outcomes (Zhang et al., 2016). The following year, five additional infections caused by ST11-KL47 MDR-hvKps carrying *bla*_KPC–2_, *bla*_CTX–M–65_, and *bla*_TEM–1_ were reported at the same hospital (Gu et al., 2018). Recently, China reported cases of ST11-KL47 and ST11-KL64 hypervirulent, carbapenem-resistant *K. pneumoniae* types, which caused liver abscess (Yang et al., 2020). There have also been reports of MDR-hvKps in other parts of Asia and throughout the world (Tang et al., 2020; Wyres et al., 2020a; Dong et al., 2022). In 2012, a case was identified in France caused by ST86-KL2 MDR-hvKp carrying *bla*_CTX–M–3_ in a patient who only resided in France and Algeria (Surgers et al., 2016). The following year, a death caused by ST23 MDR-hvKp carrying *bla*_KPC–2_ (Cejas et al., 2014) was reported in Argentina. In 2016, the United States experienced a case caused by ST23-KL1 carrying *bla*_SHV–36_ in a patient who had traveled to South America (Karlsson et al., 2019). Due to the frequent identification of MDR-hvKp in multiple countries around the world, it is obvious that this pathogen is a serious threat to global health.

In this study, we collected 76 MDR *K. pneumoniae* isolates from a tertiary hospital in Anhui, China between May 2020 and January 2022. They were characterized as harboring ESBL genes. Of these, we identified 6 isolates of MDR hvKp and 2 isolates of polymyxin-resistant ST11 *K. pneumoniae* by genomic information and phenotypic experiments. These hvKps were classified as ST11-KL64, rather than the classical ST23-KL1. We performed phenotypic experiments, whole genome sequencing, and a comparative genome analysis of these strains to determine the genetic background and evolutionary mechanisms of ST11 MDR-hvKp.

## Materials and methods

### Bacterial strains and growth conditions

A total of 76 clinical MDR *K. pneumoniae* isolates were collected from different patients between May 2020 and January 2022 (Supplementary Table 1). Isolates were plated on blood agar plates and cultured at 37°C for 24 h to isolate the bacterial clones. A VITEK 2 Compact System (bioMérieux, France) was used to identify the positive culture strains. All isolates were stored in 40% (v/v) glycerol broth at –80°C until use. A total of 4,972 isolates of *K. pneumoniae* in China as of 2021 were collected from the NCBI database (including whole genome sequences and whole genome shotgun sequences).^1^

### Determination of minimum inhibitory concentration

Antibiotic susceptibility of all strains was performed by broth microdilution as recommended by the Clinical and Laboratory Standards Institute (CLSI). *K. pneumoniae* was cultured overnight in LB liquid medium at 37°C and 220 rpm. 0.5 μL of *K. pneumoniae* solution was streaked onto LB plates and incubated at 37°C for 24 h. Several monoclonal strains were selected to adjust the concentration of the bacteria in MH (Mueller-Hinton Broth) medium (Wiegand et al., 2008). The final inoculum size for broth dilution was 5 × 10^5^ colony-forming units (CFUs)/well, after inoculating into MH medium with various concentrations of Polymyxin B. Each concentration gradient was divided into three parallel groups and grown at 37°C and 220 rpm with shaking for 24 and 48 h. The experiment was repeated three times independently.

### Growth curves

*Klebsiella pneumoniae* growth curves were established in LB medium manually. Overnight cultures were diluted to an OD_600_ of 0.02 and grown in 96-well plates at 37°C and 220 rpm with shaking. The absorbance of the culture solution at 600 nm (OD_600_) was measured every 0.5 h until it had peaked and was flat.

### Mucoviscosity assay

*K. pneumoniae* viscosity was determined using the string test (Shon et al., 2013). Strains that formed strings 5 mm or longer after stretching with the tip of a sterile inoculation loop were considered to have a hypermucoviscosity phenotype. *K. pneumoniae* was cultured overnight in LB liquid medium at 37°C and 220 rpm. The cultures were diluted the following day to an OD_600_ of 1 and centrifuged at 2,350 g for 5 min, and the OD_600_ of the supernatant was measured every minute.

### Galleria mellonella infection model

The virulence of *K. pneumoniae* isolates was evaluated using the *G. mellonella* infection model. Larvae (0.3–0.4 g) were stored in the dark and used within 3 days after shipment (Tianjin Huiyude Biotechnology Co., Ltd.). Prior to injection, the bacterial pellet was washed with sterile saline and diluted to 1 × 10^8^ CFU/mL. Using a 1 mL insulin syringe (Shanghai Kindly Ent Dev), 10 μL of the bacterial suspension was injected into the center of the second gastropod of the larvae. A group of 10 larvae were randomly selected for injection. Each treatment was performed in triplicate with 30 larvae. After injection, the larvae were incubated at room temperature and survival was monitored daily for 3 days. Death was recorded when the larvae no longer responded to touch. The larvae without or injected with 10 μL sterile saline were used as negative controls. In all cases, no dead larvae were observed in the negative control groups.

### Human whole blood survival

Peripheral blood was collected from healthy volunteers (the author himself) and stored in heparin sodium anticoagulant tubes. Logarithmic phase *K. pneumoniae* was collected and suspended in PBS at 10^8^ CFU/mL. Then, 10 μL of bacteria and 90 μL of blood were mixed in a 96-well plate and incubated for 1 h at 37°C in 5% CO_2_. After 1 h, the blood cells were lyzed using cell lysis buffer (10 mM EDTA, 0.25% Triton-X100 in PBS) and plated on TSB agar. The following day, the surviving bacterial cells were determined using a CFU assay.

### Isolation and bactericidal assay of human neutrophils

Human neutrophils were isolated from the venous blood of healthy volunteers using a human peripheral blood neutrophil isolation kit (Solarbio, China) according to the manufacturer’s instructions (Gao et al., 2019). Logarithmic phase *K. pneumoniae* was collected and suspended in PBS at 10^8^ CFUs/mL. Then, 20 μL of bacteria were mixed with 100 μL of neutrophils (10^6^ cells/ml) in a 96-well plate. The total volume was adjusted to 150 μL with PBS and they were incubated for 1 h at 37°C in 5% CO_2_. Neutrophils were lyzed after 1 h using cell lysis buffer and plating on TSB agar. The following day, the surviving bacterial cells were assessed by CFU assay.

### Whole genome sequencing, assembly, and annotation

A total of 39 isolates were sequenced, 7 isolates were sequenced by the third-generation sequencing, and the remaining 32 isolates were sequenced by the second-generation sequencing. Briefly, whole genome sequencing of *K. pneumoniae* was performed using a PacBio RS II and an Illumina HiSeq 4000 platform at the Beijing Genomics Institute (BGI, Shenzhen, China). Four SMRT cells Zero-Mode Waveguide arrays for sequencing were used with the PacBio platform to generate the subread set. PacBio subreads (length < 1 kb) were removed. The Pbdagcon program^2^ was used for self-correction. Draft genomic unitigs, which are uncontested groups of fragments, were assembled using the Celera Assembler against a high-quality corrected circular consensus sequence subreads set. To improve the accuracy of the genomic sequences, GATK^3^ and SOAP tool packages (SOAP2, SOAPsnp, SOAPindel) were used to make single-base corrections. *De novo* hybrid assembly of short Illumina reads and long PacBio reads was performed using Unicycler v0.4.8 (Wick et al., 2017) and annotated using the rapid prokaryotic genome annotation tool, Prokka 1.14.6 (Seemann, 2014). The plasmid map was drawn using BRIG 0.95 and Easyfig 2.2.5 (Alikhan et al., 2011; Sullivan et al., 2011).

### Genome profiling and comparative genomics analysis

Acquired antimicrobial resistance genes (ARGs) were identified using ABRicate version 1.0.1^4^ by aligning the genomic sequences to the ResFinder database and NCBI database (Zankari et al., 2012). The virulence factors of the isolates were identified using Kleborate and ABRicate by aligning the genomic sequences to the VFDB database (Zankari et al., 2012; Wyres et al., 2016a). Multilocus sequence typing (MLST) was performed by MLST 2.1^5^ (Jolley and Maiden, 2010). Capsule typing was performed by Kleborate (Wyres et al., 2016b; Lam et al., 2021). The HarvestTools kit (Parsnp, Gingr, and HarvestTools) and BacWGSTdb were used to perform a comparative genomic analysis and phylogenetic analysis on the different isolates and to construct phylogenetic trees based on Single nucleotide polymorphisms (SNPs) in all isolates using the maximum likelihood method. The interactive tree of life (iTOL) v5^6^ was used to draw a phylogenetic tree (Treangen et al., 2014; Letunic and Bork, 2019; Feng et al., 2021). SNPs were called from the completed genome of 21,072,329 using Snippy^7^ and the genes in which SNPs were located were annotated and functionally classified using eggNOG 5.0 (Huerta-Cepas et al., 2019). Whole genome sequencing data were deposited in the NCBI database^8^ and are publicly available under BioProject: PRJNA669320, PRJNA685326, PRJNA823907, and PRJNA838703.

### Statistical analyses

All analyses were performed using Prism software (GraphPad Software, La Jolla, CA, United States). Error bars represent SEM. All experiments were repeated at least three times.

## Results

### ST11 *Klebsiella pneumoniae* isolates were the most prevalent and exhibit hypervirulence characteristics

We performed MLST and capsular typing tests on 76 MDR isolates (Supplementary Table 1), they were resistant to at least three antimicrobial classes (Magiorakos et al., 2012). And all isolates were resistant to cefuroxime axetil, cefuroxime sodium, and levofloxacin (Supplementary Table 2). A total of six MDR-hvKp isolates were detected (Supplementary Table 3) and they were resistant to ceftriaxone, amoxicillin/clavulanic acid, ceftazidime, cefoperazone/sulbactam, cefuroxime axetil, cefuroxime, ertapenem, cefepime, cefoxitin, imipenem, levofloxacin, trimethoprim/sulfamethoxazole, piperacillin/tazobactam. Four isolates that were resistant to polymyxins included two ST11 isolates [FRPDR and 21,091,025, the minimum inhibitory concentration (MIC) of polymyxin were 16 and 8 μg/mL, respectively]. Of these, ST11 was the most abundant with 15 isolates, followed by ST15 and ST307 with 13 and 10 isolates, respectively (Supplementary Table 4). Of the ST11 isolates, nine were KL64, five were KL47, and one was KL15. All ST15 isolates were KL19, whereas all ST307 isolates were KL102. We also identified an isolate which was a single locus variant of ST11, KP2000557 (ST751-KL64). Compared with ST11, it contained a mutation in the 1,716 DNA sequence of *rpoB* (c. 1716T > G, p. Ile572Met), suggesting that it may be derived from an ST11 mutation.

**FIGURE 1:**
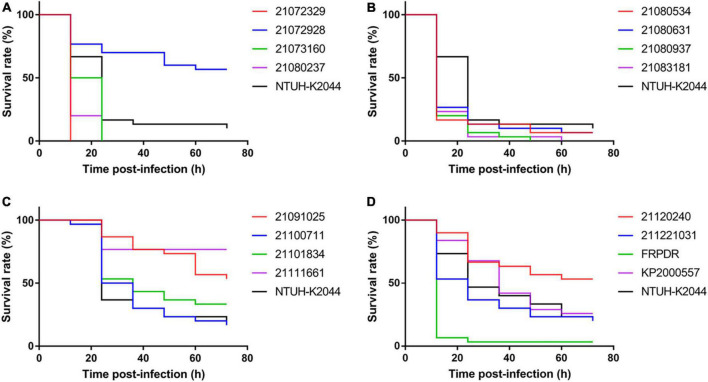
ST11 isolates showed high virulence against *Galleria mellonella*. Using the *G. mellonella* infection model to evaluate virulence of ST11 *K. pneumoniae* isolates (1 × 10^6^ CFU). **(A–D)** Are the *G. mellonella* infection model results of ST11 *K. pneumoniae* isolates.

**FIGURE 2:**
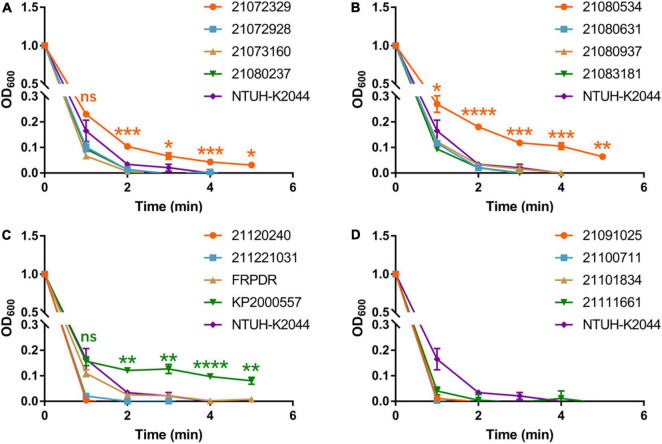
ST11 *K. pneumoniae* isolates exhibit characteristics of hypermucoviscosity. Viscosity levels of the isolates were determined by the OD_600_ of the supernatant obtained after centrifugation of the overnight culture at 2,350 g for 5 min. **(A–D)** Are the mucoviscosity assay results of ST11 *K. pneumoniae* isolates. *P < 0.05, **P < 0.01, ***P < 0.001, ****P < 0.0001.

To evaluate the virulence of these isolates and to identify hvKp, we performed growth curves, *Galleria mellonella* infection model, and mucoviscosity assay (Walker et al., 2019) using NTUH-K2044 (the first classical hypervirulent *K. pneumoniae*) as a hvKp control (Wu et al., 2009). NTUH-K2044 exhibited the highest growth rate (Supplementary Figure 1). The growth rate of the ST307 isolates was similar to that of NTUH-K2044, that of the ST11 and ST15 isolates were lower compared with that of NTUH-K2044. Eleven ST11 isolates exhibited high lethality toward *G. mellonella* and were similar or more lethal than NTUH-K2044 (Figure 1). However, only two ST15 isolates and one ST307 isolate had higher lethality compared with NTUH-K2044 against *G. mellonella* (ST15: 21,073,066, 21,091,216; ST307: 21,072,123) (Supplementary Figure 2). The OD_600_ of the isolates was adjusted to 1 and centrifuged at 2,350 *g* for 5 min (Walker et al., 2019). The supernatant of the classical hvKp NTUH-K2044 was completely transparent after centrifugation for 3 min (Figure 2 and Supplementary Figure 3), whereas that of 21,072,329, 21,080,534, and KP2000557 remained opaque, and it was difficult to centrifuge them completely (Figures 2A–C). However, in the string test, only KP2000557 could form strings above 1 cm. Nonetheless, we identified *K. pneumoniae* isolates carrying *rmpA* and *rmpA2* and having a higher or similar lethality to *G. mellonella* than NTUH-K2044 as hvKp. All 6 isolates of MDR-hvKp were ST11 isolates; thus, we focused our study on ST11. We performed human whole blood survival assays and human neutrophil bactericidal assays. Of note, 10^6^ CFU of MDR-hvKp KP2000557 did not survive in healthy human whole blood but resisted human neutrophil killing (Supplementary Figure 4). 10^6^ CFU of 21,072,928, 21,111,661, and KP2000557 were unable to survive in healthy human whole blood.

### Phylogenetic analysis of multidrug-resistant ST11 *Klebsiella pneumoniae*

**FIGURE 3:**
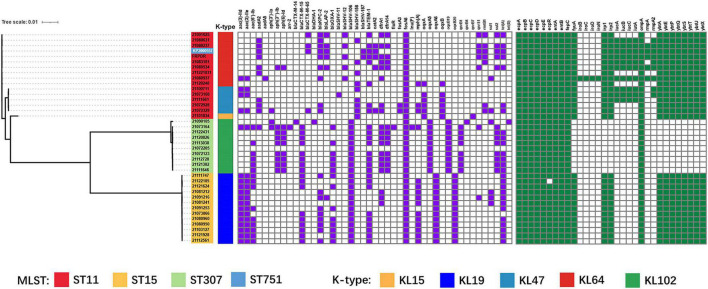
Phylogenetic tree based on core genome single nucleotide polymorphisms (SNPs) and distribution of resistance genes, virulence factors of *K. pneumoniae* isolates (ST11, ST15, ST307). Isolates with different MLST and capsule types are shown in different colors using iTOL v4 (Letunic and Bork, 2019). Carrying resistance genes are colored in purple, and carrying virulence factors are colored in green. Scale bar indicates the number of nucleotide substitutions per site.

Whole genome sequencing revealed a total of 414 resistance genes in 39 isolates (ST11, ST15, and ST307) (Figure 3). The ESBL gene that was most frequently detected and carried by each isolate was *bla*_SHV_, with 43 in total (*bla*_SHV–12_: 8, *bla*_SHV–106_: 23, *bla*_SHV–158_: 9, *bla*_SHV–187_: 3). Other detected ESBL genes included *bla*_CTX–M_ (*bla*_CTX–M–14_: 1, *bla*_CTX–M–15_: 17, *bla*_CTX–M–65_: 4). In addition to ESBL genes, other β-lactamase encoding genes were also detected including *bla*_TEM_ (*bla*_TEM–1_: 25), *bla*_KPC_ (*bla*_KPC–2_: 18), *bla*_OXA_ (*bla*_OXA–1_: 14), *bla*_*LAP*_ (*bla*_LAP–2_: 6), *bla*_DHA_ (*bla*_DHA–1_: 1) and *bla*_SHV–11_ (*bla*_SHV–11_: 1) (Liakopoulos et al., 2016). The *rmpA* and *rmpA2* were only detected in ST11 isolates and were located on plasmids. Five ST11 isolates carried both *rmpA* and *rmpA2* (21,080,237, 21,080,534, 21,080,937, FRPDR, and KP2000557). All isolates (ST11 and ST15), except ST307, carried *ybt*, which encodes yersiniabactin. Only MDR-hvKp 21080937 carried *iro*, which encodes salmochelin. The *iuc* encoding aerobactin was only detected in ST11 isolates and all *rmpA*-carrying isolates carried *iuc*. The *clb* encoding colibactin was absent in all isolates (Supplementary Table 5).

### Characterization of extended spectrum β-lactamase, carbapenemase *Klebsiella pneumoniae* carbapenemase-2 and hypervirulence plasmids carried by ST11 multidrug-resistant-hypervirulent *Klebsiella pneumoniae*

**FIGURE 4:**
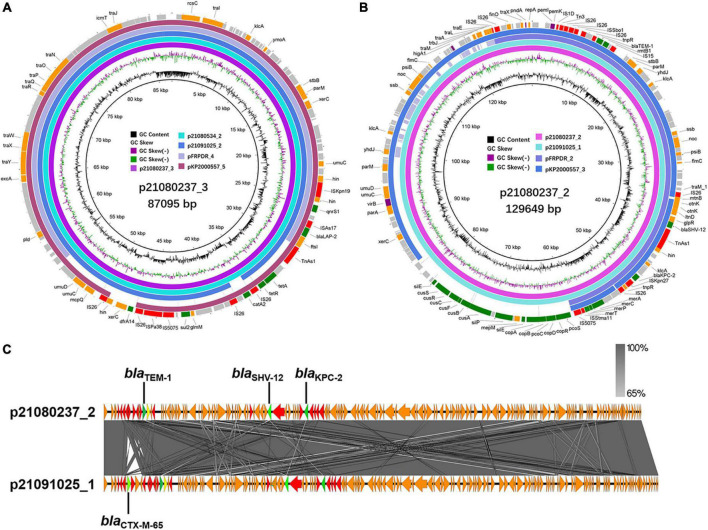
Alignment of MDR plasmids in ST11 MDR-hvKps. **(A)** Alignment of plasmid p21080237_3, plasmid p21080534_2, plasmid p21091025_2, plasmid pFRPDR_4, and plasmid pKP2000557_5 using the BLAST Ring Image Generator (BRIG 0.95) (Alikhan et al., 2011). **(B)** Alignment of plasmid p21080237_2, plasmid p21091025_1, plasmid pFRPDR_2 and plasmid pKP2000557_3. **(C)** Alignment of plasmid p21080237_2 and p21091025_1 using Easyfig 2.2.5 (Sullivan et al., 2011). ORFs encoding transposases are colored in red, and drug resistance genes are colored in green.

The generation of most MDR hvKp is due to the strains acquiring hypervirulence genes and MDR genes. These genes can be spread between strains via plasmids. Therefore, we analyzed the surrounding sequences of hypervirulence genes and MDR genes (ESBL, carbapenemase-encoding genes, and other β-lactamase encoding genes) carried by MDR hvKp and their plasmids. We performed third-generation sequencing on the 6 MDR-hvKp isolates and two polymyxin-resistant isolates. IncR plasmid p21072329_1 carried *bla*_CTX–M–65_, *bla*_KPC–2_, and *bla*_TEM–1_ (Supplementary Figure 5A). p21080237_2 and p21091025_1 belonged to the IncFII/IncR plasmid and carried *bla*_KPC–2_, *bla*_SHV–12_, and *bla*_TEM–1_ (Supplementary Figures 5B,C). pFRPDR_2 belonged to the IncFII plasmid and carried *bla*_CTX–M–65_, *bla*_KPC–2_, *bla*_SHV–12_, and *bla*_TEM–1_ (Supplementary Figure 5D). pKP2000557_3 belonged to the IncFII/IncR plasmid and carried *bla*_CTX–M–65_, *bla*_KPC–2_, *bla*_SHV–187_, and *bla*_TEM–1_ (Supplementary Figure 5E). In addition, with the exception of 21,080,937, each isolate also carried a plasmid containing *bla*_*LAP*–2_ (Figure 4A and Supplementary Figure 6). All isolates carried three sequences surrounding *bla*_TEM–1_, namely *bla*_TEM–1_ alone (p21080237_2), other drug resistance genes-*bla*_CTX–M–65_-*bla*_TEM–1_ (p21072329_1), and *bla*_CTX–M–65_-*bla*_TEM–1_ (p21091025_1, pFRPDR_2, pKP2000557_3) (Figure 4B and Supplementary Figure 7A). All isolates had *bla*_KPC–2_ co-existing with the surrounding ISs. All isolates had *bla*_SHV–12_ co-existing with upstream TnAs1. And some isolates had *bla*_KPC–2_ appearing upstream of *bla*_SHV–12_ (p21080237_2, p21080534_1, p21091025_1, pFRPDR_2) (Supplementary Figure 7B). Chromosomally located *bla*_SHV_ appeared to be site-specific, with sequences inserted downstream of the *lac* operon (Supplementary Figure 7C). The *bla*_*LAP*–2_ of all isolates formed the structure of TnAs1-*ftsI*- *bla*_*LAP*–2_ –IS*As17*-*qnrS1*-*hin*-IS*Kpn19* (Supplementary Figure 7D).

**FIGURE 5:**
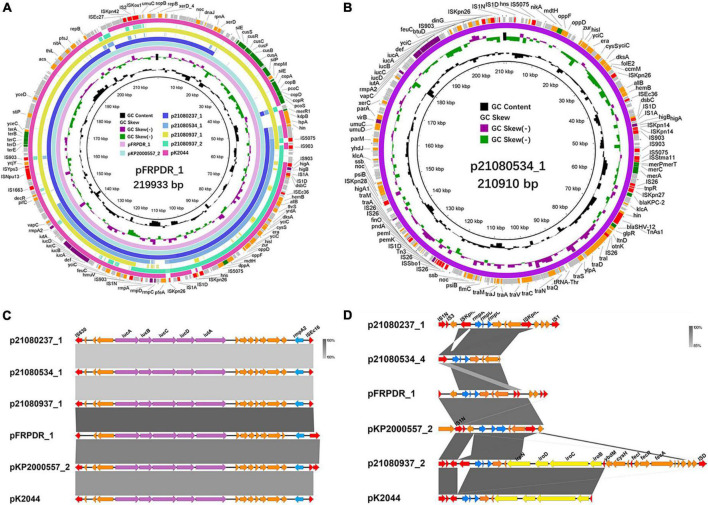
Alignment of hypervirulence plasmids in ST11 MDR-hvKps and comparison of *rmpA*, *rmpA2* gene clusters in different MDR-hvKps. **(A)** Alignment of plasmid p21080237_1, plasmid p21080534_1, plasmid p21080937_1, plasmid p21080937_2, plasmid pFRPDR_1, plasmid pKP2000557_2, and plasmid pK2044 (GenBank Accession No. AP006726) using the BLAST Ring Image Generator (BRIG 0.95) (Alikhan et al., 2011). **(B)** Plasmid map of MDR and hypervirulence plasmid p21080534_1. **(C)** Comparative analysis of *rmpA2* surrounding genes of MDR-hvKps, plotted using Easyfig 2.2.5 (Sullivan et al., 2011). **(D)** Comparative analysis of *rmpA* surrounding genes of MDR-hvKps.

The sequences around *rmpA2* carried by all isolates were completely identical, namely IS*630*-*iucA*-*iucB*-*iucC*-*iucD*-*iutA*-*rmpA2*-IS*Ec16* (Figures 5A–C). Meanwhile, the RmpA2 of all isolates were identical (Supplementary Figure 8A). The *rmpA* on p21080937_2 inserted a guanine (G) into the 286th nucleotide sequence, resulting in a frameshift mutation of *rmpA* (Supplementary Figure 8B). And a missense mutation (418C > A, Leu140Ile) occurred in *rmpA* on pKP2000557_2. There were mainly two types of sequences around *rmpA* in each isolate, the presence or absence of the downstream salmochelin operon *iro* (Figure 5D). By comparing the gene clusters of different isolates, we found that the original *rmpADC* gene cluster was *rmpA*-*rmpC*-*rmpD*-*iroN*-*iroD*-*iroC*-*iroB*. Because of the insertion of IS*Kpn26* into *iroN*, the sequence of the gene cluster was changed, resulting in the insertion mutation. ISs-mediated transfer may lead to *rmpADC* detachment from *iroBCDN*, resulting in *rmpADC* gene cluster sequences on p21080237_1, 21080534_4, pFRPDR_1 and pKP2000557_2 (Supplementary Figure 9).

Through comparison, we found that some plasmids carried by hvKp have extremely high similarity. Compared with p21080237_2, p21091025_1 carried an ESBL gene, *bla*_CTX–M–65_, which was absent from p21080237_2 (Figure 4C). NCBI database comparison revealed that p21072329_3 exhibited highly similar to the pMTY12128 from Japan (Supplementary Figure 10A). pMTY12128 had only an additional insertion of IS*Kpn8* than p21072329_3; otherwise, they share 100% homology. Furthermore, p21072329_4 shared 100% homology with p21080237_6 (Supplementary Figure 10B). All isolates, except 21,080,237, contained a ColRNAI plasmid, p21072328_5 (5,596 bp), which carries genes of unknown function (Supplementary Figure 11C).

### Phylogenetic analysis of ST11 *Klebsiella pneumoniae*

To determine the relatedness of our isolates to other Chinese isolates, we collected 4,973 whole genome sequences of all *K. pneumoniae* isolates from China contained in the NCBI database as of 2021. Of these isolates, 2,524 (50.75%) ST11 isolates were detected. We conducted a comparative genomics analysis of the ST11 and ST751 isolates (Supplementary Figure 12A). Fifteen ST11 isolates and one ST751 isolate constituted three clades based on the phylogeny which also corresponds to the K-types (Supplementary Figure 12B). The lowest SNP among all ST11 strains was 119 (21,080,237 and 21,080,631) and the highest was 3,797 (21,072,329 and 21,101,834) (Supplementary Figure 13). 21,080,237 and 21,080,631 shared high homology and they belonged to the same clade as the isolates from Zhejiang. 21,080,534 and 21,083,181 were similar to the isolates from Nanjing (KPN 02 and KPN08, etc.).

### Single nucleotide polymorphism analysis revealed possible hypervirulence and polymyxin resistance mutations

Among the MDR-hvKps, only 21,072,329 showed a hypervirulence and hypermucoviscosity phenotype without *rmpADC* and *rmpA2*. Furthermore, we did not detect *mcr*-like genes, such as *mcr-1*, in the genome or plasmid of the ST11 polymyxin-resistant isolates, FRPDR, and 21,091,025. In addition, *mgrB* had no IS insertion.

**FIGURE 6:**
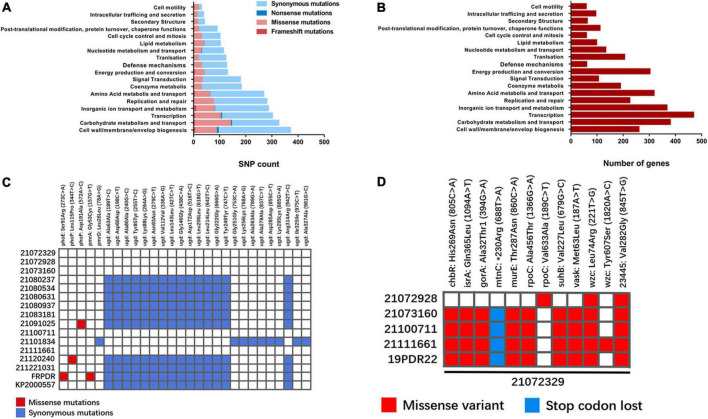
Mutational analysis of ST11 *K. pneumoniae* isolates. **(A)** Using 2107239 as the reference genome, enrichment analysis of COG categories for genes mutated in ST11 *K. pneumoniae* isolates. Different types of mutations are represented by different colors (Huerta-Cepas et al., 2019). **(B)** Distribution of functional genes annotated in 21072329 by eggNOG 5.0. **(C)** Mutations in polymyxin-related genes in ST11 *K. pneumoniae* isolates. Missense mutations are shown in red and nonsense mutations are shown in blue. **(D)** Differences in SNPs shared by 21072329 with other ST11-KL47 *K. pneumoniae* isolates.

Using 20,172,329 as a reference genome, we identified 6,283 non-repetitive mutations. Of these, 5,508 mutations were in coding sequences (CDS), 773 mutations were in non-coding regions, and 2 mutations were in tRNA coding regions. Among the mutations in the CDS, there were 4,015 synonymous mutations, 1,390 missense mutations, 64 frameshift mutations, 30 nonsense mutations, and 4 intragenic mutations. The putative functions of these mutated CDSs were classified using the eggNOG database (Figure 6A). Although “cell wall/membrane/envelop biogenesis” exhibited the highest number of mutations, most of the mutations were synonymous mutations (276) and there were only 83 missense mutations, less compared with “carbohydrate metabolism and transport” (138) and “transcription” (99). In addition, there were many genes related to “cell wall/membrane/envelop biogenesis” (260) (Figure 6B), which does not indicate that this function is prone to mutation.

Isolates 21,091,025 and FRPDR did not carry *mcr*-like genes, we hypothesized that the mutation may be located in the chromosome-mediated polymyxin resistance genes *arnT*, *eptA* (*pmrC*), *mgrB*, *phoPQ*, *pmrAB*, *pmrD*, and *ugd*. The SNPs of colistin-related resistance genes were counted and 28 SNPs were found, including 4 missense mutations and 24 synonymous mutations (Figure 6C). In 21,091,025, the 572nd nucleotide of *phoP* was mutated from adenine (A) to cytosine (C), resulting in a mutation of the 191st amino acid of the encoded PhoP from Asp to Ala. Both *phoE* and *pmrA* of FRPDR were mutated. The 273rd nucleotide of *phoE* is mutated from cytosine (C) to adenine (A), causing the mutation of the 91st amino acid of its encoded PhoE from Ser to Arg. The 157th nucleotide of *pmrA* is a guanine (G) mutated to a thymine (T), resulting in a mutation of the 53rd amino acid of PmrA encoded by Gly to Cys. These mutations may lead to polymyxin resistance. In addition, the 115th amino acid of PhoP of 21,120,240 was also mutated (Leu115Pro), however, this did not result in polymyxin resistance. We analyzed the SNPs of 21,072,329 to explore the reasons for its hypervirulence. *K. pneumoniae* with different capsule types differs greatly in the gene cluster. Thus, we only analyzed the mutations present in 21,072,329 compared with other ST11-KL47 isolates, including ST11-KL47 *K. pneumoniae* 19PDR22 that we previously obtained (He et al., 2022). Through a comparison of different ST11-KL47 isolates, we found two SNPs that were present only in 21,072,329 and located in *wzc* (221T > G, Leu74Arg) and gene *23445* (845G > T, Gly282Val) (Figure 6D). Wzc is a tyrosine kinase involved in capsular polysaccharide (CPS) synthesis and assembly, and *23445* encodes the LysR family transcriptional regulator. However, it remains to be determined whether their mutation leads to the generation of a hypervirulence phenotype.

## Discussion

It is generally considered that highly virulent pathogens, including hvKp, rarely develop resistance to antibiotics. Thus, the higher the virulence, the weaker the resistance. The emergence of MDR-hvKp challenges this idea. Since the twenty-first century, hvKps have gradually spread globally, resulting in significant medical and health issues. In 2015, the first MDR-hvKp was identified in China, and its MLST was primarily ST23 (Zhang et al., 2016). Subsequently, China reported a variety of other MLST MDR-hvKps, in particular, ST11 (Gu et al., 2018; Yang et al., 2020; Zheng et al., 2020). According to the existing research (Russo et al., 2018), we identified hvKp carrying both *rmpA* and *rmpA2* in this study. Among the 76 MDR *K. pneumoniae* isolates we collected, 16 represented ST11 and its variants, 5 were ST11 MDR-hvKps, and one was ST11 variant MDR-hvKp. These MDR-hvKps were resistant to at least 13 of the 16 tested antibiotics and carried at least two β-lactamases and seven resistance genes. These MDR-hvKps exhibited hypermucoviscosity and hypervirulence. However, the results of the string test showed that only KP2000557 could form strings above 1 cm, which was inconsistent with the results of the mucoviscosity assay. Some studies have shown that the string test is not a method that can accurately identify hvKp, and it may be better to identify hvKp with multiple methods. In addition, patients with MDR-hvKp 21,080,237 and 21,080,534 were both around 30 years old. This indicates that the most dangerous MLST of *K. pneumoniae* in China remains ST11, and it could be evolved from an MDR-KP to an MDR-hvKp, which poses a serious threat to public health.

Except for the classical ST23-KL1 hvKp, most of the hvKps of other MLSTs exhibit hypervirulence phenotypes resulting from the acquisition of hypervirulence plasmids (Yang et al., 2021). The generation of ST11 MDR-hvKps is caused by the transfer of multidrug resistance and hypervirulence plasmids (Xu et al., 2021). We detected *rmpA* and *rmpA2* in 5 isolates of MDR-hvKp and they were all located in hypervirulence plasmids. RmpA2 was identical in all strains, but there were differences in RmpA. The surrounding sequences of *rmpA* on these hypervirulence plasmids are quite different; however, *rmpA2* shows less of a difference. In addition, *iucABCD* and *iutA* are always downstream of *rmpA2*. Some isolates have *iroBCDN* downstream of *rmpA*, whereas some isolates have only ISs. Upon comparison, the reason for the deletion of *iroBCDN* downstream of *rmpA* could be the insertion of an IS into *iroN*, causing a mutation in *iroN*. Moreover, ISs mediate the transfer of *rmpA*, which separates *rmpA* from *iroBCDN*. The initially reported hypervirulence plasmids, pK2044 and pLVPK, carried *iroBCDN*, *iucABCD*, *iutA*, *rmpA*, and *rmpA2* (Chen et al., 2004; Chou et al., 2004). We speculated that the hypervirulence plasmid may have evolved from pK2044 and pLVPK with the insertion of ISs.

We found that some plasmids showed only minor differences, including differences in SNPs, deletions, or IS insertions. This suggests the existence of transfers between plasmids. With the exception of 21,080,237, we found a ColRNAI plasmid with unknown function in other isolates. This plasmid only carried *mbeB*, *mbeB*, *mobC*, the gene encoding toxin, and many genes of unknown function. This plasmid may be related to bacterial plasmid mobilization and adaptability, but its detailed function needs to be determined. Of note, we found that the IncFII/IncR plasmid, p21080534_1 of MDR-hvKp 21,080,534, carried the carbapenemase gene (*bla*_KPC–2_), ESBL gene (*bla*_SHV–12_), hypervirulence genes (*iucABCD*, *iutA*, and *rmpA2*), and mercury resistance genes (*merACPT*). This is a rare plasmid carrying both hypervirulence and multidrug resistance genes, which may be derived from the hypervirulence plasmid and IS carrying multidrug resistance genes. Besides p21080534_1, other plasmids carrying both hypervirulence genes and multidrug resistance genes have been recently reported in China (Shen et al., 2019). These plasmids facilitate the transformation of cKp into MDR-hvKp and pose unprecedented challenges in controlling the spread of MDR-hvKp.

Polymyxins were once considered the last line of defense against MDR Gram-negative bacteria (Li et al., 2006); however, with the continuous abuse of antibiotics, bacteria have developed a variety of polymyxin resistance mechanisms, including plasmid-mediated colistin resistance gene *mcr-1*, two-component system PhoP/Q, PmrA/B, and *pmrHFIJKLM* operon (Groisman, 2001; Gunn, 2008; Olaitan et al., 2014; Liu et al., 2016). ST11 isolates, FRPDR (MDR-hvKp) and 21,091,025, are resistant to polymyxin, but we did not find *mcr*-like genes. Moreover, we found missense mutations in *phoP* (21,091,025: 572A > C, Asp191Ala), *phoE* (FRPDR: 273C > A, Ser91Arg), and *pmrA* (FRPDR: 157G > T, Gly53Cys) on chromosome 21,091,025. The mutation of the 53rd amino acid of PmrA has been reported (Sun et al., 2009) and we speculate that these mutations may be responsible for polymyxin resistance. We did not detect *rmpA* or *rmpA2* in 21,072,329, which only carried one siderophore (yersiniabactin). Interestingly, 21,072,329 showed a phenotype of hypervirulence and hypermucoviscosity, and its virulence and viscosity were higher compared with NTUH-K2044. By aligning the genome of 21,072,329 with other isolates, we found two unique SNPs (wzc: 221T > G, Leu74Arg, *23445*: 845T > G, Val282Gly). Wzc is involved in the synthesis and assembly of *K. pneumoniae* CPS. The hyperviscous phenotype of hvKp is largely due to overproduction of CPS. Therefore, the mutation of wzc may cause 21,072,329 to exhibit the characteristics of hvKp. Gene *23445* encodes the LysR family transcriptional regulator. It cannot be ruled out that there are other factors that lead to its hypervirulence and hypermucoviscosity phenotype, and could also be related to an epistatic phenomenon with a combination of mutations. This study still has some limitations, namely a single medical institution was sampled over a short time period. Thus, the results of this study need to be further corroborated. Therefore, the results of this study need to be further confirmed by constructing mutant genes in cKp or constructing reverse mutations in 21,072,329.

In conclusion, we performed a genome-wide analysis of ST11, ST15, and ST307 that broke out in Hefei, Anhui, especially ST11 MDR-hvKp. We identified the mechanisms of plasmid transmission for ST11 MDR-hvKp and the correlation between isolates. In addition, we also reported the dangerous hypervirulence plasmid, p21080534_1, which not only carried the hypervirulence genes, *rmpA2, iucABCD*, and *iutA*, but also the ESBL gene, *bla*_SHV–12_, and the carbapenemase gene, *bla*_KPC–2_. Currently, the spread of hypervirulence and multidrug resistance plasmids carried by *K. pneumoniae* may have exceeded our expectations. It is urgent to limit the spread of MDR-hvKp and their associated plasmids.

## Data availability statement

The datasets presented in this study can be found in online repositories. The names of the repository/repositories and accession number(s) can be found below: https://www.ncbi.nlm.nih.gov/, PRJNA669320; https://www.ncbi.nlm.nih.gov/, PRJNA685326; https://www.ncbi.nlm.nih.gov/, PRJNA823907; https://www.ncbi.nlm.nih.gov/, PRJNA838703.

## Ethics statement

Ethical review and approval were not required for the study on human participants in accordance with the local legislation and institutional requirements. Written informed consent for participation was not required for this study in accordance with the national legislation and the institutional requirements.

## Author contributions

BS and ZH designed the project. ZH, WX, and HZ performed the experiments. ZH, YD, HL, LZ, and CZ collected isolates. ZH analyzed the data and wrote the manuscript. YL and BS critically revised the manuscript. All authors read and approved the final manuscript.

## Footnotes

1
https://www.ncbi.nlm.nih.gov/pathogens/

2
https://github.com/PacificBiosciences/pbdagcon

3
https://www.broadinstitute.org/gatk/

4
https://github.com/tseemann/abricate

5
https://cge.food.dtu.dk/services/MLST/

6
http://itol.embl.de/

7
https://github.com/tseemann/snippy

8
https://www.ncbi.nlm.nih.gov/bioproject/
