# Corrigendum: Epidemiological characteristics an outbreak of ST11 multidrug-resistant and hypervirulent *Klebsiella pneumoniae* in Anhui, China

**DOI:** 10.3389/fmicb.2023.1231407

**Published:** 2023-06-13

**Authors:** Zhien He, Weifeng Xu, Hang Zhao, Wei Li, Yuanyuan Dai, Huaiwei Lu, Liping Zhao, Changfeng Zhang, Yujie Li, Baolin Sun

**Affiliations:** ^1^Department of Oncology, The First Affiliated Hospital of USTC, Division of Life Sciences and Medicine, University of Science and Technology of China, Hefei, Anhui, China; ^2^College of Life Science and Technology, Mudanjiang Normal University, Mudanjiang, China; ^3^Department of Clinical Laboratory, Anhui Provincial Hospital of Anhui Medical University of China, Hefei, China; ^4^Clinical Laboratory Center, First Affiliated Hospital, Anhui University of Traditional Chinese Medicine, Hefei, China

**Keywords:** extended-spectrum β-lactamase, *Klebsiella pneumonia*, multidrug-resistant and hypervirulent *Klebsiella pneumoniae* (MDR-hvKp), virulence plasmid, whole genome sequencing

In the published article, there was an error in affiliations 1 and 2. Instead of “^1^Department of Oncology, The First Affiliated Hospital, University of Science and Technology of China, Hefei, China, ^2^School of Life Sciences and Medicine, University of Science and Technology of China, Hefei, China,” it should be “^1^Department of Oncology, The First Affiliated Hospital of USTC, Division of Life Sciences and Medicine, University of Science and Technology of China, Hefei, Anhui, China.”

The authors apologize for this error and state that this does not change the scientific conclusions of the article in any way. The original article has been updated.

