# Supplementary material for: Epidemiological characteristics an outbreak of ST11 multidrug-resistant and hypervirulent Klebsiella pneumoniae in Anhui, China
Source: Front Microbiol. 2022 Sep 23;13:996753. doi: 10.3389/fmicb.2022.996753 (PMC9537591; doi:10.3389/fmicb.2022.996753)
Supplement: Supplementary file 1 [file Data_Sheet_1.DOCX]

Supplementary Material

## Supplementary Figures

**
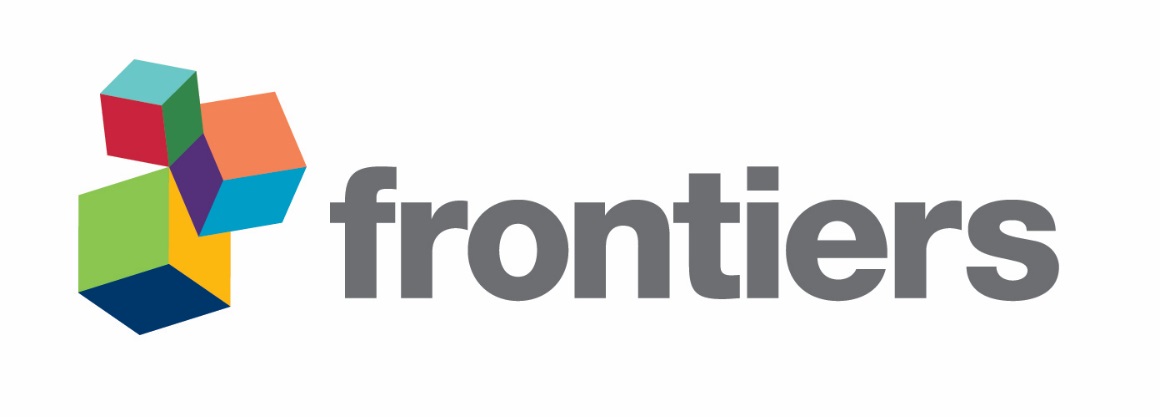
**


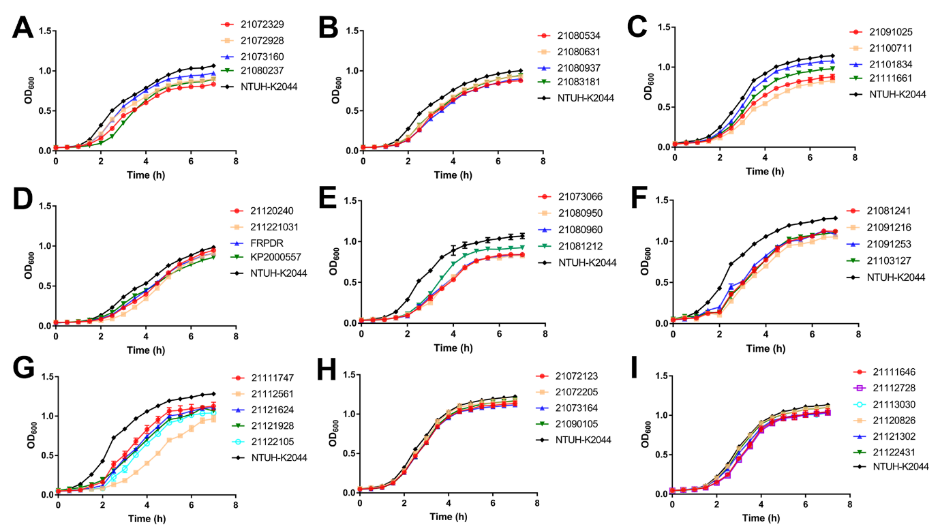


**Supplementary Figure 1.** Growth curves of the collected isolates.

(A), (B), (C) and (D) are the growth curves of ST11 *K. pneumoniae* isolates. (E), (F) and (G) are the growth curves of ST15 *K. pneumoniae* isolates. (H) and (I) are the growth curves of ST307 *K. pneumoniae* isolates.


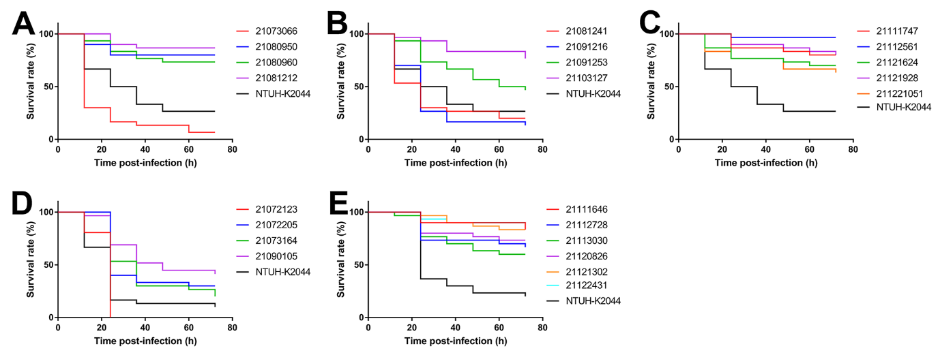


**Supplementary Figure 2.** *Galleria mellonella* infection model assay of ST15 *K. pneumoniae* isolates and ST307 *K. pneumoniae* isolates.

(A), (B) and (C) are the *G. mellonella* infection model assay results of ST15 *K. pneumoniae* isolates. (D) and (E) are the *G. mellonella* infection model assay results of ST307 *K. pneumoniae* isolates.


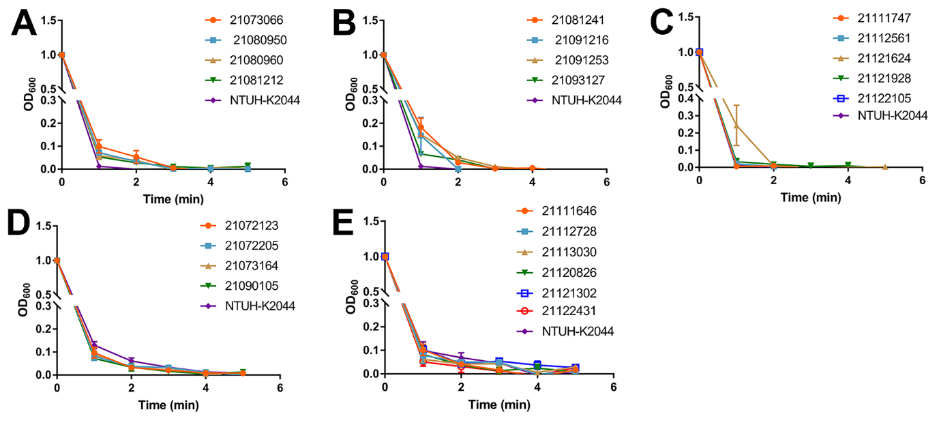


**Supplementary Figure 3.** Mucoviscosity assay of ST15 *K. pneumoniae* isolates and ST307 *K. pneumoniae* isolates.

(A), (B) and (C) are the mucoviscosity assay results of ST15 *K. pneumoniae* isolates. (D) and (E) are the mucoviscosity assay results of ST307 *K. pneumoniae* isolates.


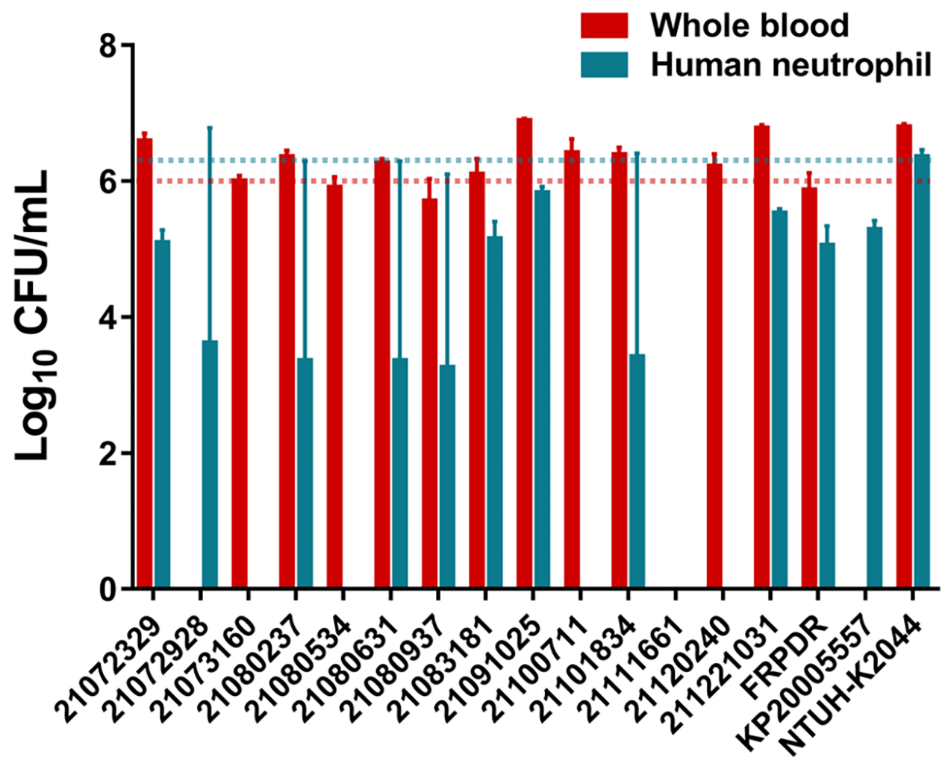


**Supplementary Figure 4.** Human whole blood survival and human neutrophil bactericidal assay of ST11 *K. pneumoniae* isolates.

Human whole blood survival assay results are shown in red, and human neutrophil bactericidal assay results are shown in blue. The red dotted lines represents the amount of bacteria initially added in the human whole blood survival assay (1 × 10^6^ CFU), and the blue dotted lines represents the amount of bacteria initially added in the human neutrophil bactericidal assay (2 × 10^6^ CFU).


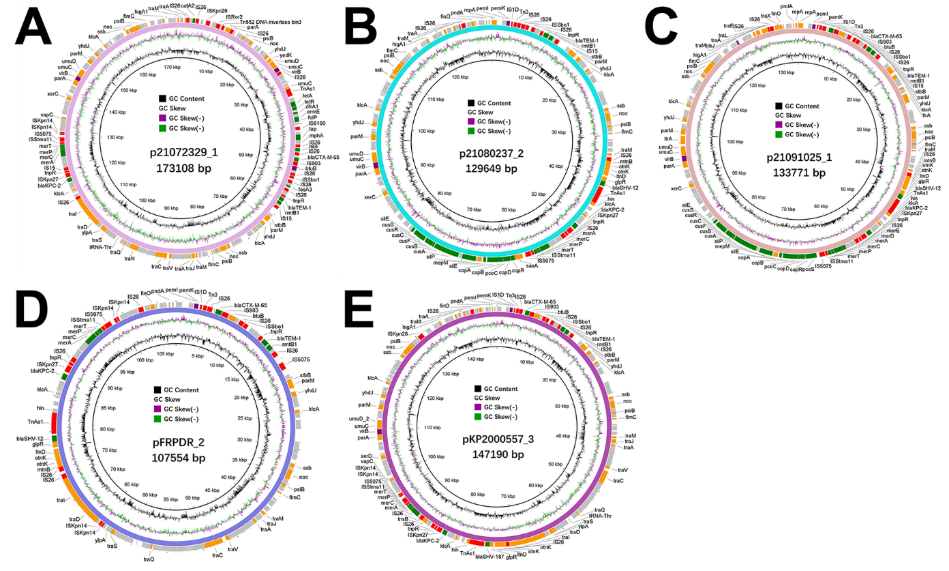


**Supplementary Figure 5.** Plasmid maps of plasmids carrying the ESBL genes in MDR-hvKps.

(A) Plasmid map of p21072329_1, using the BLAST Ring Image Generator (BRIG 0.95). (B) Plasmid map of p21080237_2. (C) Plasmid map of p21091025_1. (D) Plasmid map of pFRPDR_2. (E) Plasmid map of pKP2000557_3.


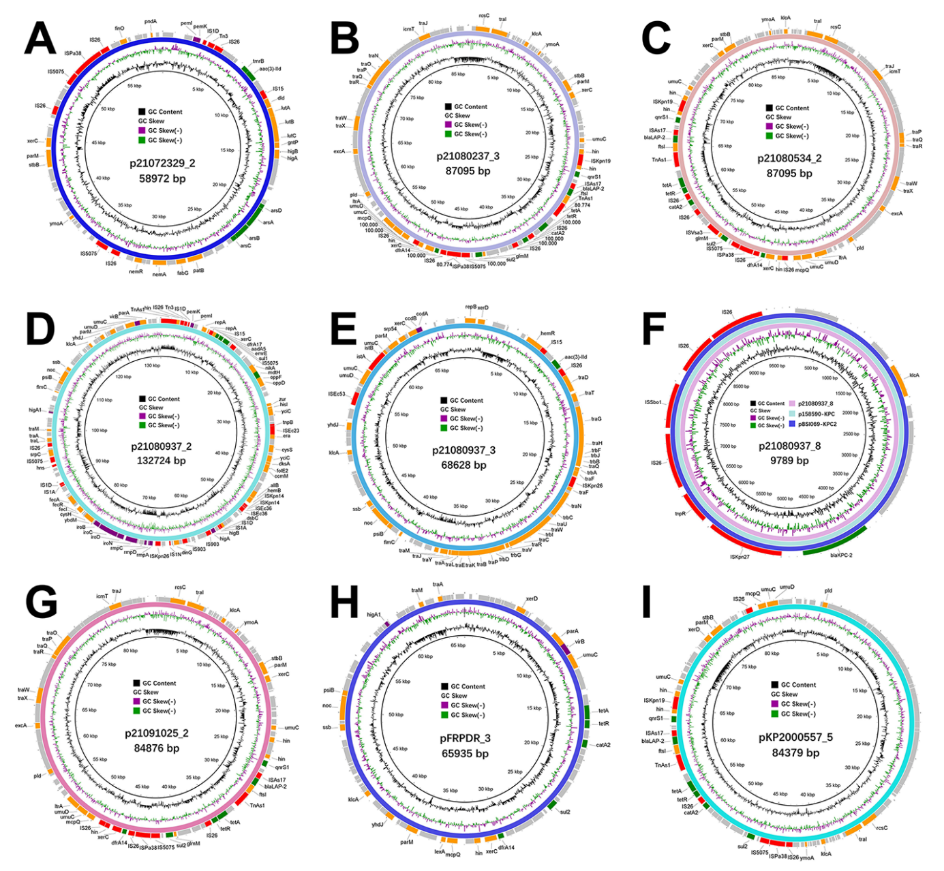


**Supplementary Figure 6.** Plasmid maps of plasmids carrying resistance genes in MDR-hvKps.

(A) Plasmid map of p21072329_2, using the BLAST Ring Image Generator (BRIG 0.95) (Alikhan et al., 2011). (B) Plasmid map of p21080237_3. (C) Plasmid map of p21080534_2. (D) Plasmid map of p21080937_2. (E) Plasmid map of p21080937_3. (F) Plasmid map of p21080937_8. (G) Plasmid map of p21091025_2. (H) Plasmid map of pFRPDR_3. (I) Plasmid map of pKP2000557_5.


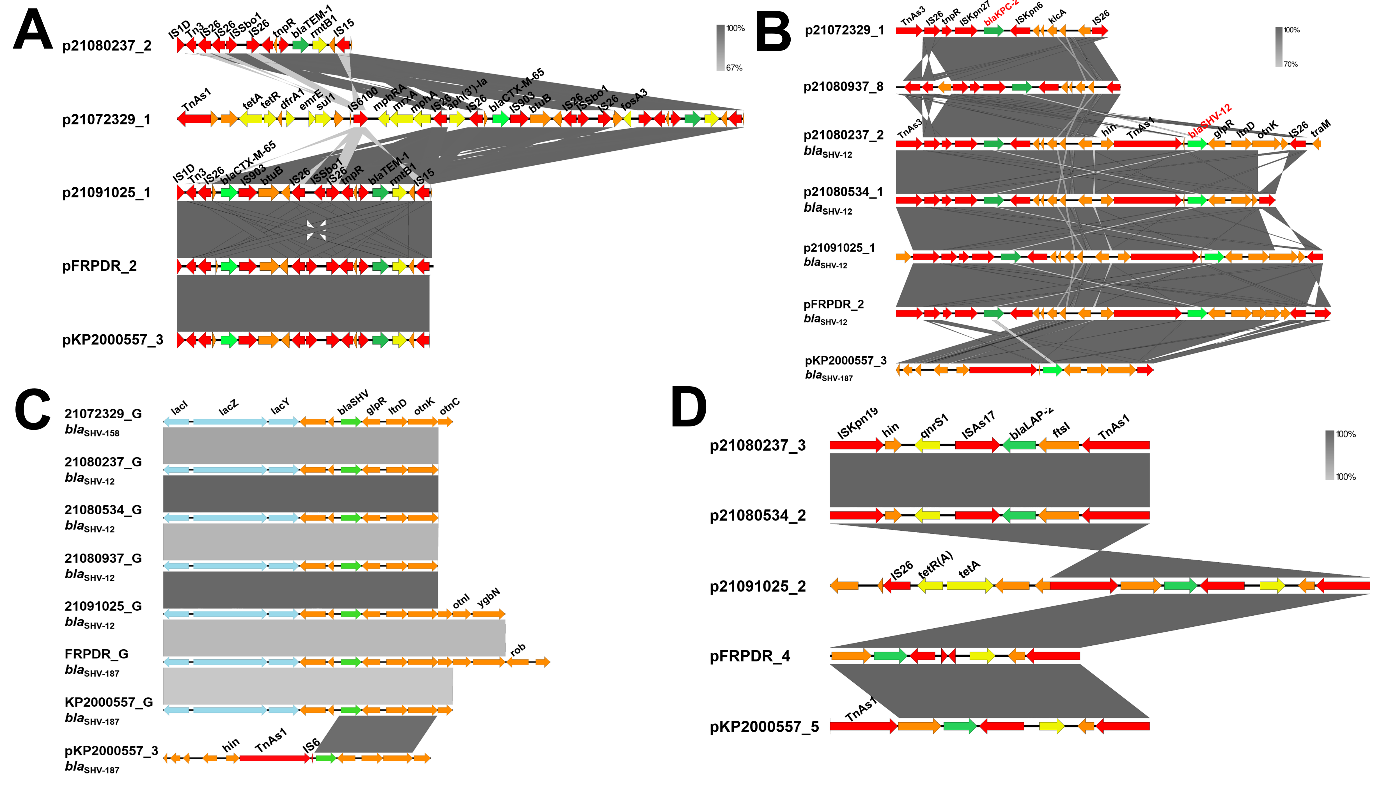


**Supplementary Figure 7.** Plasmid maps of plasmids carrying hypervirulence genes in MDR-hvKps.

(A) Comparative analysis of *bla*_CTX-M_ *and bla*_TEM_ surrounding genes of MDR-hvKps, plotted using Easyfig (Sullivan et al., 2011). ORFs encoding transposases are colored in red. (B) Comparative analysis of *bla*_KPC_ surrounding genes of MDR-hvKps. (C) Comparative analysis of *bla*_SHV_ surrounding genes of MDR-hvKPs. (D) Comparative analysis of *bla*_LAP_ surrounding genes of MDR-hvKPs.


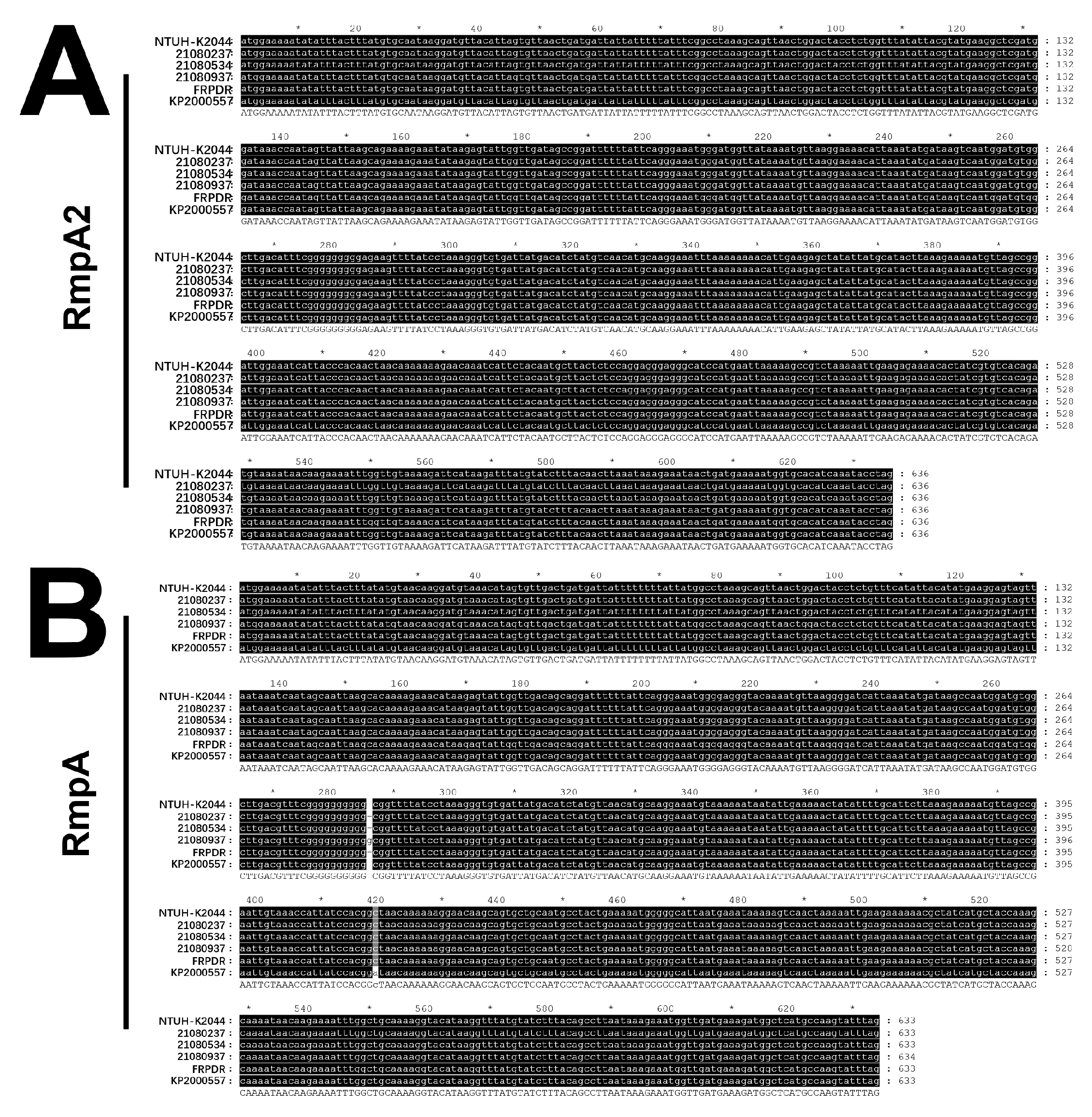


**Supplementary Figure 8.** Alignment of protein sequences of RmpA and RmpA2 from different isolates.

(A) Alignment of protein sequences of RmpA2 from different isolates. (B) Alignment of protein sequences of RmpA from different isolates.


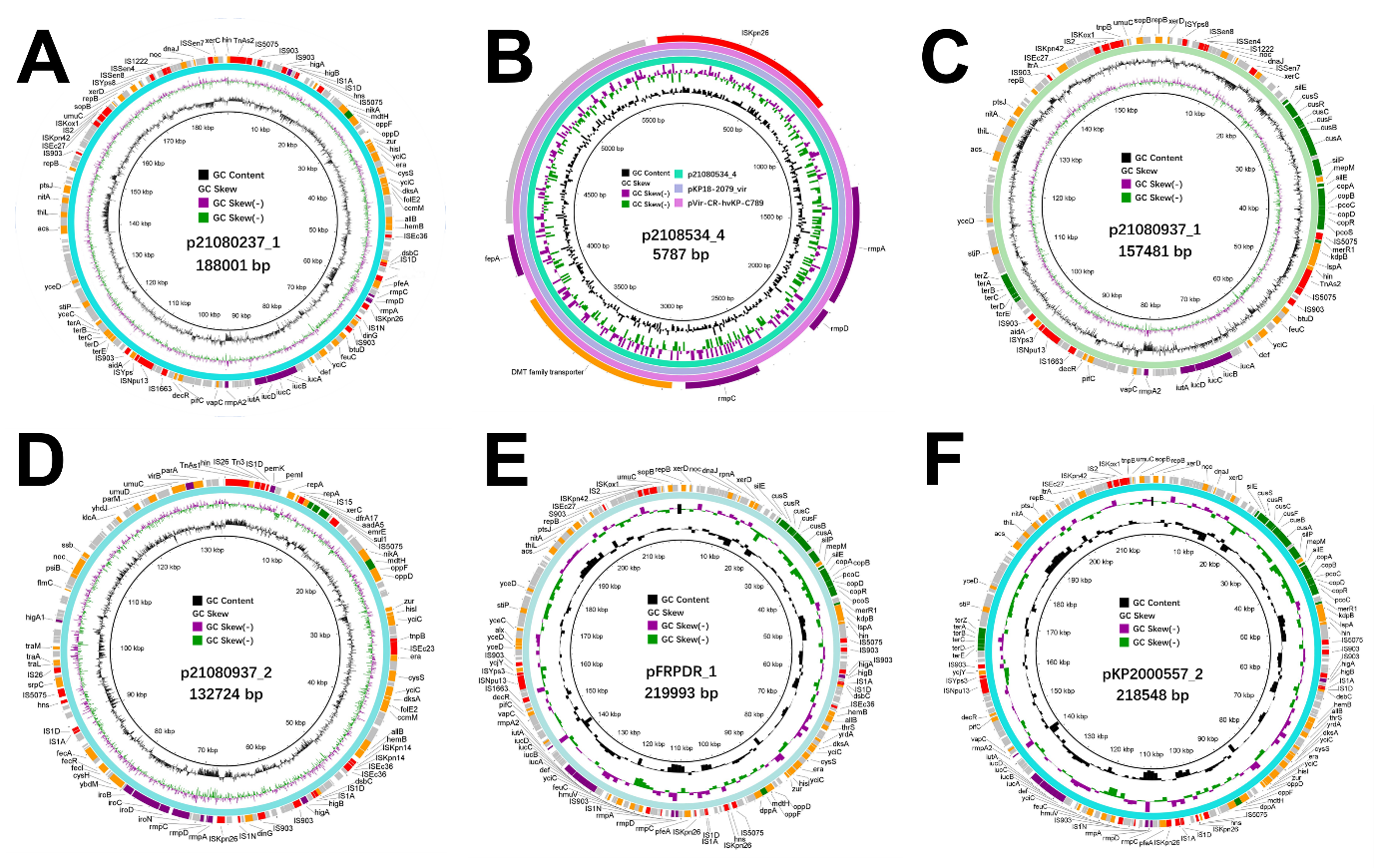


**Supplementary Figure 9.** Plasmid maps of plasmids carrying hypervirulence genes in MDR-hvKPs.

(A) Plasmid map of p21080237_1, using the BLAST Ring Image Generator (BRIG) (Alikhan et al., 2011). (B) Alignment of plasmid p21080534_4, plasmid pKP18-2079_vir (GenBank Accession No. MT090958) and pVir-CR-hvKP-C789 (GenBank Accession No. CP034416). (C) Plasmid map of p21080937_1. (D) Plasmid map of p21080937_2. (E) Plasmid map of pFRPDR_1. (F) Plasmid map of pKP2000557_2.

**
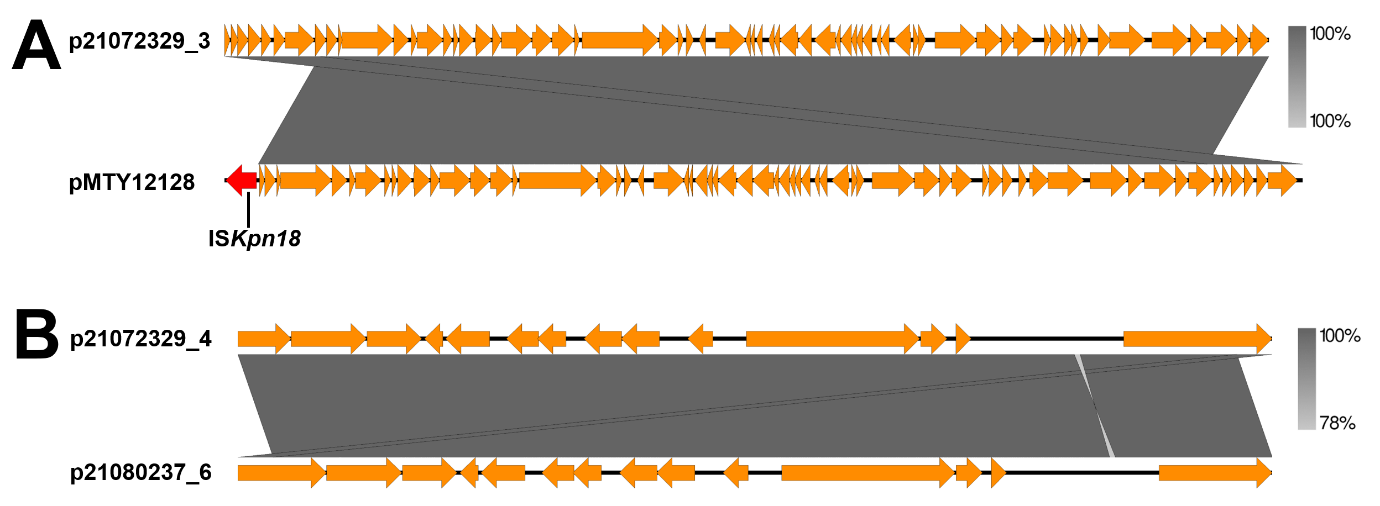
**

**Supplementary Figure 10.** Alignment analysis among plasmids with higher homology of ST11 MDR hvKps.

(A) Sequence alignment analysis of p21072329_3 and pMTY12128 (GenBank Accession No. AP024758). (B) Sequence alignment analysis of p21072329_4 and p21080237_6.

**
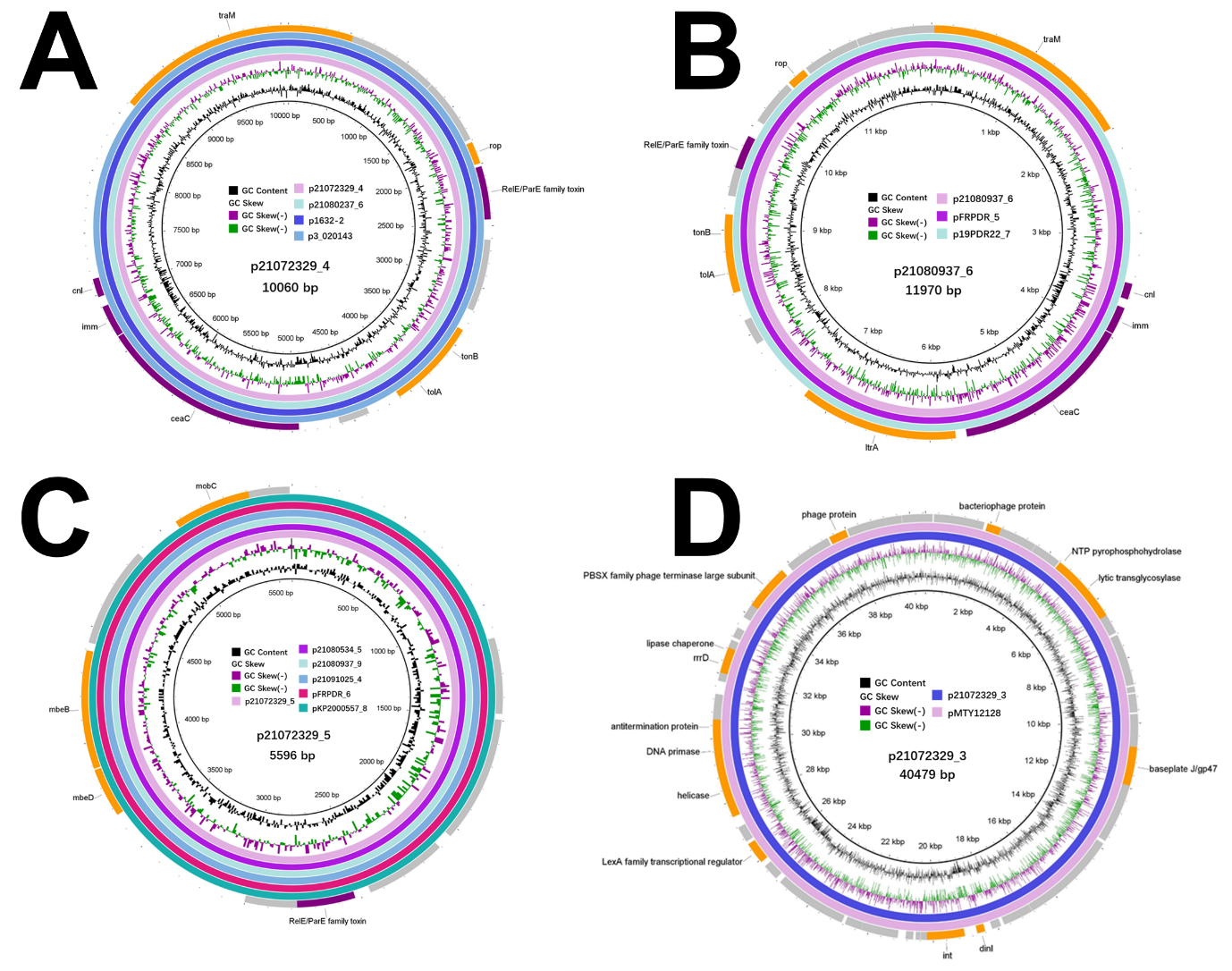
**

**Supplementary Figure 11.** Alignment of plasmids in ST11 MDR-hvKPs.

(A) Alignment of plasmid p21072329_4, plasmid p21080237_6, plasmid p1632-2 (GenBank Accession No. CP084499) and plasmid p3_020143 (GenBank Accession No. CP028545) using the BLAST Ring Image Generator (BRIG) (Alikhan et al., 2011). (B) Alignment of plasmid p21080937_6, plasmid pFRPDR_5 and plasmid p19PDR22_7 (GenBank Accession No. CP076551). (C) Alignment of plasmid p21072329_5, plasmid p21080534_5, plasmid p21080937_9, plasmid p21091025_4, plasmid pFRPDR_6 and plasmid pKP2000557_8. (D) Alignment of plasmid p21072329_3 and plasmid pMTY12128 (GenBank Accession No. AP024758).

**
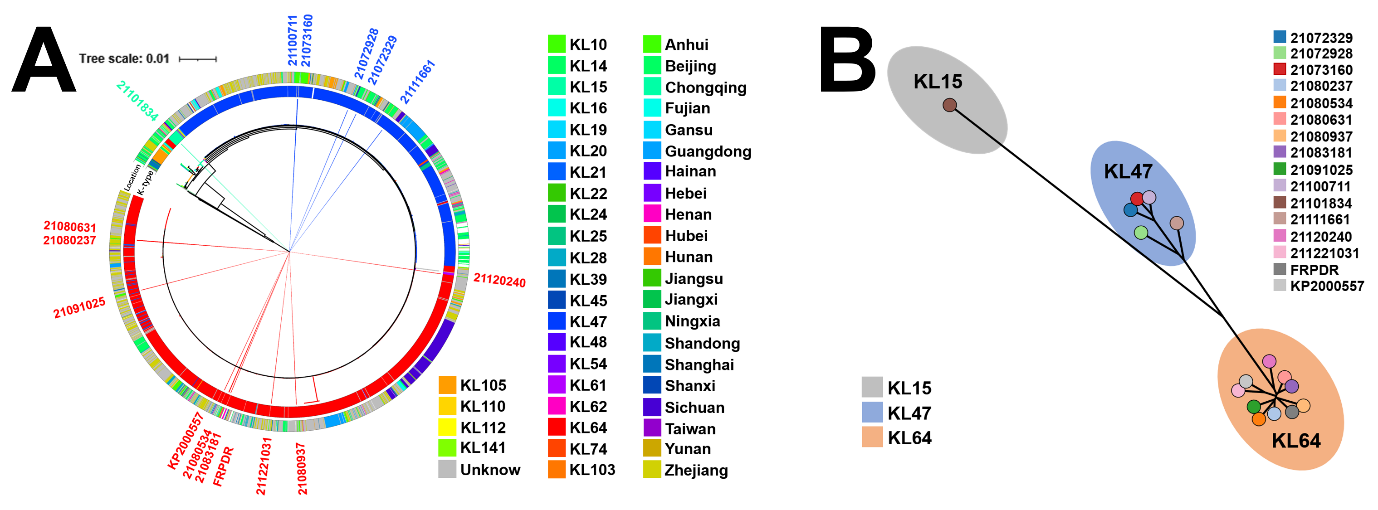
**

**Supplementary Figure 12.** Phylogenetic analysis of ST11 *K. pneumoniae* isolates.

(A) Phylogenetic analysis of the collected ST11 *K. pneumoniae* isolates and ST11 *K. pneumoniae* strains from China in the NCBI database, drawn by iTOL v4 (Letunic and Bork, 2019). Different MLST and capsule types are highlighted in different colors. Circles outside the tree indicate the MLST and geographic location of each strain. (B) Phylogenetic network of collected ST11 *K. pneumoniae* isolates based on Core Genome Multilocus Sequence Typing (cgMLST), drawn by BacWGSTdb (Feng et al., 2021). Different capsular types are highlighted in different colored backgrounds.

**
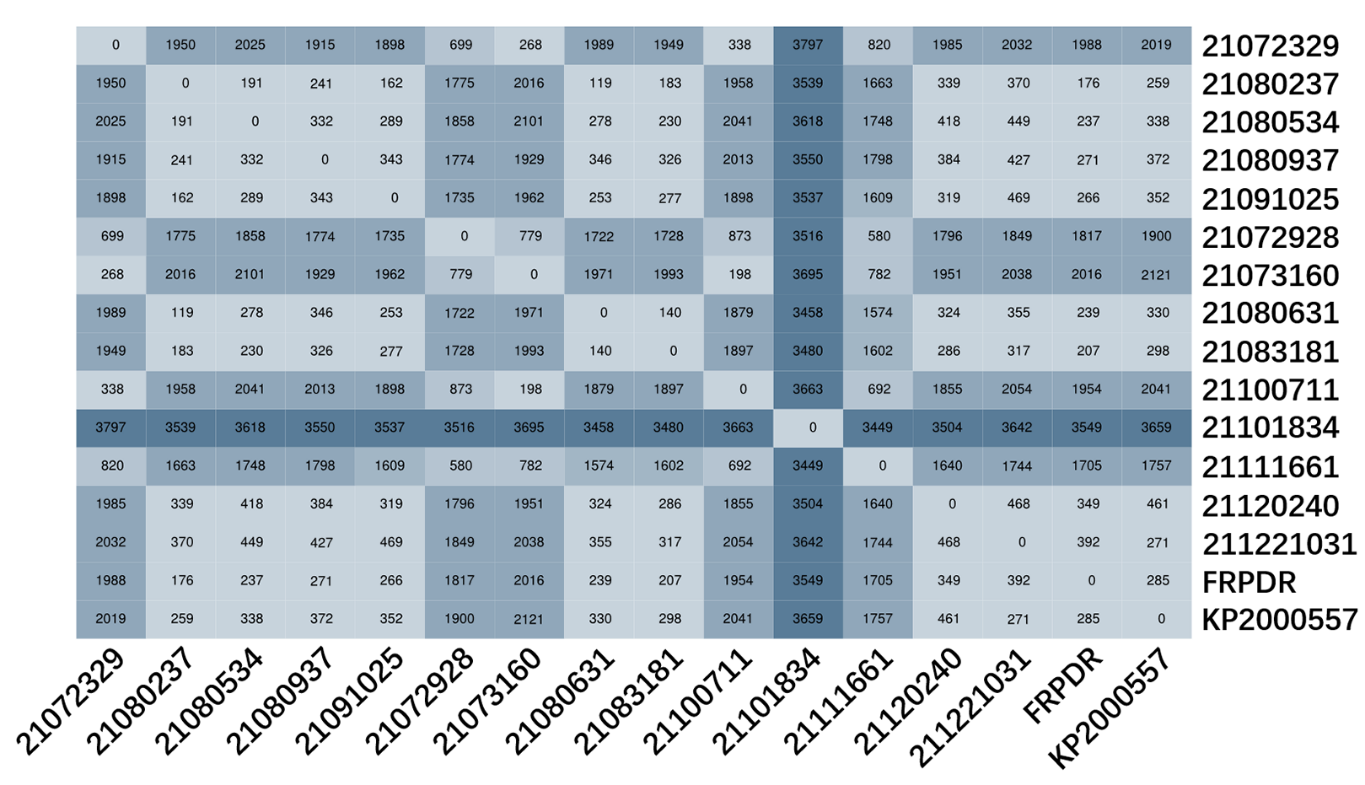
**

**Supplementary Figure 13.** Heatmap of SNP distribution of ST11 *K. pneumoniae* isolates.

Heatmap of SNP distribution of ST11 *K. pneumoniae* isolates drawn using BacWGSTdb (Feng et al., 2021), with colors deepening as SNPs increase.

## Supplementary Figures

ALIKHAN, N. F., PETTY, N. K., BEN ZAKOUR, N. L. & BEATSON, S. A. (2011). BLAST Ring Image Generator (BRIG): simple prokaryote genome comparisons. *BMC Genomics.* 12**,** 402.

FENG, Y., ZOU, S., CHEN, H., YU, Y. & RUAN, Z. (2021). BacWGSTdb 2.0: a one-stop repository for bacterial whole-genome sequence typing and source tracking. *Nucleic Acids Res.* 49**,** D644-D650.

LETUNIC, I. & BORK, P. (2019). Interactive Tree Of Life (iTOL) v4: recent updates and new developments. *Nucleic Acids Res.* 47**,** W256-W259.

SULLIVAN, M. J., PETTY, N. K. & BEATSON, S. A. (2011). Easyfig: a genome comparison visualizer. *Bioinformatics.* 27**,** 1009-10.
