# Supplementary material for: Epidemiological characteristics an outbreak of ST11 multidrug-resistant and hypervirulent Klebsiella pneumoniae in Anhui, China
Source: Front Microbiol. 2022 Sep 23;13:996753. doi: 10.3389/fmicb.2022.996753 (PMC9537591; doi:10.3389/fmicb.2022.996753)
Supplement: Supplementary file 2 [file Table_3.DOCX]

**Supplementary Table 3.** Genome information of MDR-hvKps collected in this study.

| Name | Source | Genome size(bp) | GC Content | Coding Genes | Chromosome (bp) | tRNAs | rRNAs | MLST | K-type |
| --- | --- | --- | --- | --- | --- | --- | --- | --- | --- |
| 21072329 | Sputum | 5,744148 | 57.43% | 5,544 | 5,450,821 | 87 | 25 | 11 | KL47 |
| 21080237 | Diversion fluid | 5,897,186 | 57.45% | 5,692 | 5,393,517 | 86 | 27 | 11 | KL64 |
| 21080534 | Diversion fluid | 5,776,460 | 57.32% | 5,574 | 5,422,972 | 86 | 25 | 11 | KL64 |
| 21080937 | Sputum | 5,870,673 | 57.45% | 5,659 | 5,393,376 | 85 | 25 | 11 | KL64 |
| FRPDR | Sputum | 6,029,648 | 57.28% | 5,856 | 5,557,247 | 89 | 25 | 11 | KL64 |
| KP2000557 | Blood | 6,071,809 | 57.47% | 5,888 | 4,948,687 | 87 | 25 | 751 | KL64 |
